# A school-based intervention improved dietary intake outcomes and reduced waist circumference in adolescents: a cluster randomized controlled trial

**DOI:** 10.1186/s12937-017-0299-5

**Published:** 2017-12-11

**Authors:** Angélica Ochoa-Avilés, Roosmarijn Verstraeten, Lieven Huybregts, Susana Andrade, John Van Camp, Silvana Donoso, Patricia Liliana Ramírez, Carl Lachat, Lea Maes, Patrick Kolsteren

**Affiliations:** 1 Departmento de Biociencias, Grupo Nutrición Alimentación y Salud, Facultad de Ciencias Químicas, Universidad de Cuenca, Avenida 12 de Abril y Avenida Loja, 0101168 Cuenca, Ecuador; 2 Independent researcher, Ghent, Belgium; 3 Department of Food Safety and Food Quality, Faculty of Bioscience Engineering, Ghent University, Coupure Links 653, B-9000 Ghent, Belgium; 4 Poverty, Health and Nutrition Division, International Food Policy Research Institute, 2033 K St, NW, Washington, DC USA; 5 Department of Public Health, Ghent University, Ghent, Belgium

**Keywords:** Health promotion, Dietary intake, Andes, Cluster randomized controlled trial

## Abstract

**Background:**

In Ecuador, adolescents’ food intake does not comply with guidelines for a healthy diet. Together with abdominal obesity adolescent’s inadequate diets are risk factors for non-communicable diseases. We report the effectiveness of a school-based intervention on the dietary intake and waist circumference among Ecuadorian adolescents.

**Methods:**

A pair-matched cluster randomized controlled trial including 1430 adolescents (12–14 years old) was conducted. The program aimed at improving the nutritional value of dietary intake, physical activity (primary outcomes), body mass index, waist circumference and blood pressure (secondary outcomes). This paper reports: (i) the effect on fruit and vegetable intake, added sugar intake, unhealthy snacking (consumption of unhealthy food items that are not in line with the dietary guidelines eaten during snack time; i.e. table sugar, sweets, salty snacks, fast food, soft drinks and packaged food), breakfast intake and waist circumference; and, (ii) dose and reach of the intervention. Dietary outcomes were estimated by means of two 24-h recall at baseline, after the first 17-months (stage one) and after the last 11-months (stage two) of implementation. Dose and reach were evaluated using field notes and attendance forms. Educational toolkits and healthy eating workshops with parents and food kiosks staff in the schools were implemented in two different stages. The overall effect was assessed using linear mixed models and regression spline mixed effect models were applied to evaluate the effect after each stage.

**Results:**

Data from 1046 adolescents in 20 schools were analyzed. Participants from the intervention group consumed lower quantities of unhealthy snacks (−23.32 g; 95% CI: −45.25,-1.37) and less added sugar (−5.66 g; 95% CI: −9.63,-1.65) at the end of the trial. Daily fruit and vegetable intake decreased in both the intervention and control groups compared to baseline, albeit this decrease was 23.88 g (95% CI: 7.36, 40.40) lower in the intervention group. Waist circumference (−0.84 cm; 95% CI: −1.68, 0.28) was lower in the intervention group at the end of the program; the effect was mainly observed at stage one. Dose and reach were also higher at stage one.

**Conclusions:**

The trial had positive effects on risk factors for non-communicable diseases, i.e. decreased consumption of unhealthy snacks. The program strategies must be implemented at the national level through collaboration between the academia and policy makers to assure impact at larger scale.

**Trial registration:**

ClinicalTrial.gov-NCT01004367.

## Background

In the last decades (since 1980 until 2013), overweight and obesity prevalence has increased around 47% among children and adolescents; affecting both high and low- and middle-income countries [1]. Overweight-obese children show higher mortality rates and are more likely to become obese adults [2]. The risk of obesity and other non-communicable diseases (NCDs) is attenuated by reducing blood pressure, increasing physical activity, supporting diets rich in fruit, vegetables, grains and nuts, and restricting the intake of sugared beverages and sweets [3–7]. In Ecuador, type 2 diabetes, hypertension and stroke are leading causes of death [8], with a larger disease burden in the urban areas [9]. Overweight, obesity and dyslipidemia [10], together with a diet poor in fiber, fruit and vegetables, and high in added sugar, refined grains and processed food are prevalent among Ecuadorian adolescents [11].

Prevention is needed to tackle this increasing burden of NCDs [12]. Interventions focusing on education and improvements of the food environment have proven to be effective to reduce the risk of NCDs [12–14]. Schools are suitable settings to implement preventive interventions for adolescents as they deal with environmental drivers [14] with respect to dietary intake and its determinants [13]. However, school-based lifestyle interventions in LMICs have methodological and conceptual flaws i.e. they are often not theory- and evidence-based or adapted to the specific context [13]. In Latin America, only a few school-based interventions targeting healthy eating and/or physical activity have been implemented [13, 15, 16]. The available programs in the region are usually short term, target small samples and/or at risk populations [13, 16], and showed deficiencies in their designs and evaluations [13, 16, 17]. Unfortunately, school-based interventions have not been performed in Andean States such as Colombia, Bolivia, Ecuador and Perú, where the ethnic, cultural and social context differs in comparison with other Latin American countries [18, 19]. This is especially important as health promotion success rates rely on context considerations [20].

We conducted a school-based cluster randomized controlled trial with parental involvement called “ACTIVITAL”, aimed at improving the nutritional value of dietary intake and physical activity in a sample of school-going Ecuadorian adolescents. To our knowledge, the ACTIVITAL trial is innovative in its setting as it (i) was tailored to the local context using a theoretical framework and participatory approaches and (ii) targeted multiple populations (i.e. adolescents, school staff, and parents) and dietary risk factors for NCDs as well as physical activity. This paper reports: (i) the overall effect of the trial after three consecutive school years on dietary intake (primary outcome) and waist circumference, (ii) the effect of the trial after the first 17 months and the last 11 months of implementation, as well as (ii) the dose and reach of the strategies implemented. The effect on physical activity and body mass index was reported elsewhere [21].

## Methods

### Setting and context

The study targeted 12–14 year-old adolescents and was conducted in the urban area of Cuenca, the third largest city of Ecuador. At the time of the study, the Ecuadorian school system was comprised of four levels: elementary school (3–5 year-old children), primary school (1st-7th grade; 6–11 year-old children), middle school (8th to 10th grade; 12–14-year-old adolescents) and high school (1st-3rd year high school; 15–17 year-old adolescents). Within the education system, there are both private and public schools. Students attend school either in the morning (7:00–13:00) or in the afternoon (12:00–18:00), with one break of approximately 30 min. A school year in Cuenca runs from September to June. Most adolescents (73%) regularly attend school in the urban area of Cuenca [22]. All schools must follow a standard curriculum and use government issued learning materials.

*A la carte* foods (e.g. pre-packaged snacks, hot meals and soft drinks) are offered by private on-site school food kiosks. These operators function independently but are regulated by the Ministries of Health and Education. Adolescents also have easy access to street food near the schools.

### Study design and sample size

**Fig. 1:**
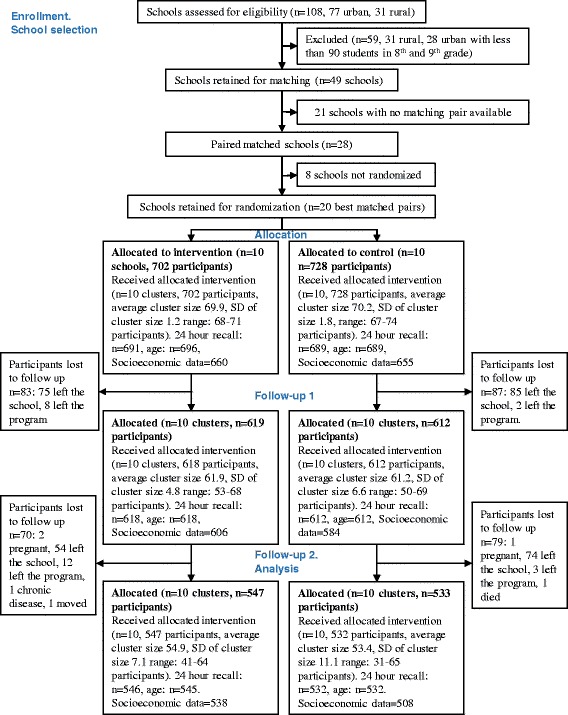
Flow chart of progress

This study was conducted in 20 schools. A pair-matched cluster randomized controlled trial was designed with the schools (clusters) as units of randomization. To reach the desired sample size of 70 students per school, schools were eligible if they (i) were located in the urban area of Cuenca, and (ii) had at least 90 students enrolled in the 8th and 9th grade. The schools’ selection process is described in Fig. 1: 28 schools were matched by size (total number of students; no more than a 15% difference), type (public-private), school gender (single gender or co-ed. schools) and socioeconomic status (monthly fee; no more than 30% difference) into 14 pairs. The best ten matching pairs were selected. From the ten allotted pairs, each school was randomly assigned to either the treatment group or the control group, using random numbers in Stata 12.0 by a researcher who was not directly involved in the implementation. Adolescents and school staff were not aware about the existence of a counterfactual school.

In each school, two 8th and two 9th grades were randomly selected. All adolescents from these grades were invited to participate and 70 students were selected at random. A total of 10 matched pairs of schools including an average sample of 65 children per school considering a Type I error of 5%, a coefficient of between-cluster variation (K_m_) of 0.15 and a power of 80% [23], allowed for the detection of a decrease in 10% of the energy percentage derived from fat. This corresponded to a total sample size of 1300 adolescents in 20 schools. Considering an expected 10% drop-out we sampled 1430 adolescents.

### Intervention development

**Fig. 2:**
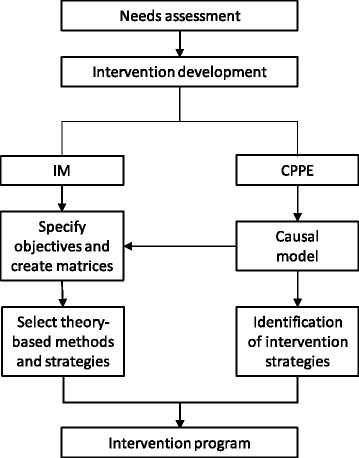
Description of the intervention development process. IM: Intervention mapping, CPPE: Comprehensive and Participatory Planning and Evaluation

Figure 2 summarizes the intervention development process. A needs assessment, including both qualitative [24] and quantitative [10, 11] data, was performed to ensure appropriateness of the program. Focus group discussions identified the following factors as influencing dietary intake: lack of knowledge about nutrition quality and healthy eating, less healthy eating habits at high school, and greater accessibility to pocket money used to buy tasty yet unhealthy food [24]. A dietary assessment prior to the study showed that the study population consumed insufficient fiber, fruit and vegetables, and an excess of added sugar, refined grains and processed foods during snacking [11]. These data served to define the intervention objectives and strategies using the Intervention Mapping (IM) [25] and the Comprehensive and Participatory Planning and Evaluation (CPPE) approach [26]. The possible intervention strategies identified during the CPPE were combined with the IM techniques to define the final intervention strategies [27]. This process resulted in the development of the intervention program named ACTIVITAL with the following intervention objectives: adolescents (i) decrease their sugar intake, (ii) increase their daily fruit and vegetable intake, (iii) decrease their unhealthy snack intake, (iv) increase their healthy breakfast intake, and (v) school food kiosks increase the offer of healthy food. Separate matrices of change objectives for adolescents, parents and school staff were generated. Finally, different effective theoretical methods were identified from literature [25, 28] to translate the objectives into intervention strategies.

### Intervention strategies and components

Intervention strategies were integrated into a curriculum-based (educational toolkits) and an environment-based (workshops and social events) component implemented in two different intervention stages (Table 1).

**Table 1:** The ACTIVITAL intervention packages, components and strategies to improve the nutritional value of dietary intake

Stage	IP	Component/Strategy	Content of the session	Responsible	Target population	Support material
Stage one	IP1	Curriculum-based component/Interactive educational toolkit: Classes every two weeks	1. Food pyramid 2. Healthy eating (introduction) 3. Healthy breakfast 4. Healthy snacking 5. Sugary drinks 6. Game. Financial autonomy	Life science school teachers or external teachers	Adolescents (8th -9th grades)	Booklets, games, didactic material
	IP1	Environment-based component/School food kiosks staff workshops	1. Identification of needs and problems 2. Prioritization of needs and problems 3. Introduction to food safety 4. HACCP and recipe development 5. Food pyramid 6. Cooking skills 7. Food preparation 8. Breakfast, snacks, sugary drink and fruit and vegetable preparation 9. National legislation, adaptation and implementation 10. Management of the food kiosk	ACTIVITAL staff	Food kiosks staff	Recipes, leaflets, books, food.
	IP1	Environment-based component/Parental workshops	1. Food pyramid 2. Breakfast, snacks and sugary drinks 3. Healthy eating and physical activity	ACTIVITAL staff	Parents	Booklets based on the curriculum based component
	IP1	Environment-based component/Preparation of a healthy breakfast	Students prepare a healthy breakfast at school in small groups	Teachers, ACTIVITAL staff	Adolescents (8th - 9th grades)	Books, food, utensils, blackboards.
Stage two	IP2	Curriculum-based component/Interactive educational toolkit: Classes every two weeks	1. Nutrients 2. Food labels 3. Portion sizes 4. Fruit and vegetables 5. Healthy lunch and dinner 6. Advertisements	Life science school teachers or external teachers	Adolescents (10th and 1st bachelor grades)	Booklets, games, didactic material
	IP2	Environment-based component/School food kiosks staff workshops	1. Portion sizes and nutritional guidelines 2. Healthy menu planning 3. Analysis, evaluation and discussion of implanting healthy menus	ACTIVITAL staff	Food kiosks staff	Recipes, leaflets, books, food.
	IP2	Environment-based component/Parental workshops	1. Portion sizes 2. Food labels and advertisements	ACTIVITAL staff	Parents	Booklets based on the curriculum based component

*HACCP* hazard analysis and critical control points, *IP* intervention package, *IP1* intervention package one (September 2010 – February 2011), *IP2* intervention package two (September 2011 – February 2012)

**Fig. 3:**
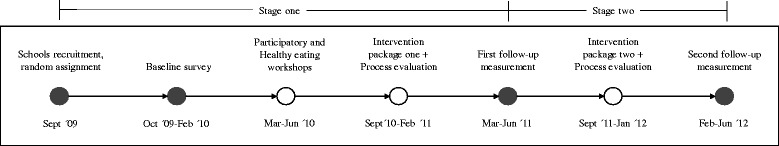
Timeline and measurements of the ACTIVITAL trial. ●Activities performed in both, intervention and control schools. ○ Activities performed in the intervention schools only

In the intervention schools, the ACTIVITAL program was implemented**.** The first stage was comprised of three activities: (i) participatory workshops with school staff and adolescents to increase ownership of the intervention and to revise the implementation strategy, (ii) introductory workshops on eating healthily with school staff and adolescents, and (iii) the implementation of intervention package one. Stage two only included the implementation of intervention package two (Table 1 and Fig. 3). In the control schools, no additional activities other than the existing national curriculum followed in health science lectures were included.

### Outcomes

The nutritional value of dietary intake was a primary outcome and included the following variables: added sugar intake, fruit and vegetables intake, unhealthy snacking in general, consumption of unhealthy school snacks and breakfast intake. Energy contribution from fat per day was also included as a primary outcome (%). Waist circumference was a secondary outcome. All outcome measurements were performed by trained health related professionals at baseline and after stage one and two, at 17 and 28 months, respectively (Fig. 3). All the outcomes are analyzed at individual level.

#### Dietary intake

Dietary intake was estimated by means of two 24-h dietary recalls conducted on two randomly chosen weekdays. The average of the 2 days was used for the final analysis to estimate mean group intake [29]. Portion sizes and food consumption were estimated using standardized local utensils (measuring cups or graduated cylinders filled with water). If detailed information was missing for ingredients and/or cooking methods, we obtained these from recipes which were prepared in triplicate by local volunteering housewives.

In the absence of an Ecuadorian food composition table, the United States (USDA, 2012), Mexican (INNSZ, 1999), Central American (INCAP/OPS, 2012) and the Peruvian (CENAN/INS, 2008) databases were searched. For data that was still unavailable, food labels and the results of local proximal analysis were used. In the case of the latter, 12 samples per food item were randomly taken from local markets and then mixed to form a composite sample. One analytical portion was analyzed in triplicate for moisture and dry matter, ash, total fat by Weibull, total nitrogen by the Kjeldahl method and total carbohydrates were determined by difference [30].

Added sugar included artificially added sugars to processed or prepared food [31]. Sugar content was unavailable in the searched food composition tables for 271 of the total 872 food items reported. Data on added sugar for these food items were obtained from food labels (*n* = 175 food items), by extrapolating the sugar content from food items with similar nutritional characteristics (*n* = 73 food items) or by using the information of the standardized recipes (*n* = 23 food items). Added sugar intake is reported as g/day.

Fruit intake included raw fruit and fruit used in juices or any other preparation. Vegetables included dark green, red and orange vegetables, either raw or boiled. Beans or starchy vegetables were not included as they were not the main target in the educational toolkit. Fruit and vegetables were classified as one food group reported as g/day consumed.

Unhealthy snacking is defined as the consumption of “unhealthy foods” eaten during snack time. Snack times - including morning, afternoon, and evening snacks - were defined according to the schools’ schedules (morning or afternoon) and the day of the week. In the case of weekdays, the times were set as follows: in the morning schools, morning snack was set between 7:00–13:00 and afternoon snack from 16:00–18:00. For afternoon schools, the timings were morning snack from 8:00–11:00 and afternoon snack from 12:00–18:00. Night snacks were set equally for the whole sample at any hour later than 21:00. The timing during weekend was similar for all the participants: morning snack from 9:00–12:00, afternoon snack from 15:00–18:00 and night snack at any hour later than 21:00. Secondly, all the “unhealthy” food items rich in sodium, fat or added sugar but low in nutrition value (usually available in vending machines, cafeterias and food kiosks [32]) consumed during these snack times were identified. These items comprised: (a) table sugar and sweets (honey, candies, chocolates, ice creams, sweet cookies, traditional sweet desserts and sugar added to juices, coffee, etc.), (b) salty snacks and fast food (packaged salty snacks, salty crackers, French fries, pizza, hamburgers) (c) soft drinks (soda, artificial sweetened juices, energy drinks) and (d) other packaged food (ketchup, packaged soups, gelatine). Finally, the intake of unhealthy foods per day during all the snack times was calculated. Unhealthy snacking is reported as g/day.

Unhealthy snacking at school was defined by identifying consumers of unhealthy foods prepared at school according to the following procedure: (i) from the list of recipes classified as unhealthy foods, we identified those prepared at school; next (ii) the participants were classified as 0 = non-consumers, if they had not reported any of the school-prepared items in the 24-h recalls, and 1 = consumers if at least one of the school-prepared item was reported in any recall.

Breakfast time was defined following a similar approach as the snack timing definition. For morning schools, breakfast time was set between 5:00–7:00 and for afternoon schools from 5:00–8:00. Breakfast intake is reported as a dichotomous variable (0 = non-consumers; 1 = consumers).

Energy from fat was calculated by dividing the daily energy of fat by the total energy intake per day (E%/day).

#### Other measurements

Waist circumference was measured in duplicate by two trained interviewers using standardized procedures. Waist circumference was measured twice at the mid-point between the last rib and the iliac-crest.

Socio-economic characteristics were assessed at baseline using the Integrated Social Indicator System tool for Ecuador [33]. Poverty was defined using the definition of Unsatisfied Basic Needs (UBN). This system categorizes a household as poor if one or more deficiencies in access to education, health, housing, water, electricity and employment are reported. Adolescents were allocated to one of two groups: ‘Poor’ if at least one deprivation was present or ‘Better-off’ if none was reported. The following extra questions based on the national census [34] about remittances and snack allowance were asked: ‘Does the family receive remittances from abroad?’ ‘Does the adolescent receive a daily snack allowance?’ and ‘How much does the adolescent receive for his/her snack allowance?’ Age was obtained from the reported date of birth.

#### Dose and reach of the intervention

Dose and reach of the intervention strategies were evaluated for different audiences (adolescents, parents and school staff) using field notes and attendance signed forms. For “dose”, the number of activities performed (i.e. classes, workshops, events) was divided by those scheduled. For “reach”, the number of participants (i.e. adolescents, parents and school staff) attending all the classes/workshops was divided by the number of invited participants.

### Data management and data analysis

Waist circumference and socio-economic data was entered in duplicate into Epidata (Epidata Association, Odense, Denmark) by two independent researchers. Any discrepancy was corrected using the original forms. Food intake data was entered using an online software designed to analyze 24-h recall data (Lucille software 0.1, 2010, Ghent University; www.foodintake.ugent.be). Data management and statistical analysis were conducted using Stata 12.0 (Stata Corporation, Texas, USA). Statistical significance was set at 5% and all tests were two-sided.

Descriptive data are reported as proportions, means with SD, or medians with 25th and 75th percentile using all the available data per follow-up time point after adjustment for the cluster design.

Outcome differences at baseline between dropout and data retained for analysis were assessed using linear regression models adjusted for treatment allocation matching pairs and cluster design. Absolute differences in outcomes at baseline between the intervention and the control group were calculated and highlighted when they were larger than 5%.

An intention-to-treat analysis was performed to evaluate the intervention effect at the end of the program and after each intervention stage. The overall effect after the full intervention period was assessed using linear mixed models. The models were adjusted for baseline differences between the treatment and the control group [35]. The models comprised the school pair and participants as random effect. The treatment allocation was nested as a random slope within each pair. Adolescent’s sex and UBN at baseline were included as fixed effects. The effect of the intervention was evaluated by testing the interaction term between follow-up time (in months) and treatment allocation. The results of the interaction term between the treatment groups with time (in months) were multiplied by 28 (duration of the trial) to calculate the overall intervention effect. Therefore, the reported differences between the intervention and the control group correspond to the effect after the whole program implementation and indicates the mean differences for continuous dependent variables and the proportion difference for dichotomous outcomes between the treatment and the control group [36]. The Akaike Information Criteria (AIC) and Schwartz’s Bayesian Information Criteria (BIC) were used to select the optimal covariance structure for the models.

As intervention stages one (17 months) and two (11 months) were considerably different in timing as well as in strategies and content (Fig. 3 and Table 1), the intervention effect was estimated separately. Regression spline mixed effects models were used for this purpose, accounting for the effects of clustering by individual and school. One knot (the point of time where the slope of the linear function changes) was defined at the mean time (in months) of the first follow-up data collection (17 months). The Stata command “mkspline” was used to create two auxiliary variables (t1 = time in months one and t2 = time in months two). The models were adjusted for baseline differences between the treatment and the control group [35] and were built with the same random effects, random slope and fixed effects as the linear mixed models. The effect of the intervention was evaluated by testing the interaction term between time1 and treatment allocation and then again time2 and treatment allocation. The Beta coefficients of these interaction terms were multiplied by 17 for the effect after the first stage and by 11 for the second stage, corresponding to the respective duration of both stages. Therefore, the reported differences between the intervention and the control group correspond to the effect after each intervention stage and indicates the mean differences for continuous dependent variables and the proportion difference for dichotomous outcomes between the treatment and the control group [36].

#### Sensitivity analysis

We assessed the influence of missing data on the outcomes with a *P* < 0.1. For this purpose, we used a multiple imputation strategy using chained equations (*n* = 50 imputations) to impute the missing outcome data under the assumption of ‘missing at random. Predictors for the regression models for the imputation were adolescent’s sex, UBN and Body Mass Index z-score at baseline.

## Results

A total of 1430 adolescents from 20 schools were recruited (Fig. 1). There were relatively more girls than boys (66% vs. 59%) in the intervention group (Table 2). More participants in the intervention group received remittances from abroad (24% vs. 17%). Outcome data at baseline and the two follow-up periods are reported in Table 3. At baseline, adolescents from the intervention group consumed (7%) more fruit and vegetables than those in the control group (median (IQR) intake: 204.6 g (119.1–337.0) for the intervention and 191.5 (104.1–304.2) for the control group). Whilst adolescents in the control group consumed 11% more unhealthy foods during snacking (median (IQR) intake: 94.5 g (27.5–220.0) for the intervention and 97.5 g (40.0–258.0) for the control group).

**Table 2:** Baseline characteristics at individual and cluster level^a^

	Intervention group		Control Group	
Individual level	n	Mean ± SD or %	n	Mean ± SD or %
Age (y)	696	12.9 ± 0.8	692	12.9 ± 0.8
Girls (%)	702	66.2	699	58.2
Better off (%)	670	68.2	655	67.5
Remittances from abroad (%)	643	23.8	636	16.5
Snack allowance (%)	651	82.5	643	82.9
Amount of snack allowance ($)	642	0.9 ± 0.6	653	0.9 ± 0.6
Education of the father (years)	568	12.1 ± 4.8	571	12.2 ± 4.9
Education of the mother (years)	611	11.8 ± 4.7	613	11.5 ± 4.8
Cluster level	n	Median (25th–75th) or %	n	Median (25th–75th) or %
Students per school (n)	10	751 (335–1169)	10	787 (326–1335)
Public schools (%)	10	50.0	10	50.0
Co-ed. schools (%)	10	70.0	10	70.0
Monthly fee ($)	10	7.00 (0.0–68.3)	10	6.50 (0.0–81.8)
Morning schools (%)	10	60.0	10	60.0

^a^Summary statistics adjusted for the cluster design

**Table 3:** Primary and secondary outcomes at baseline and at follow-up measurements by treatment group^a^

	Measurement	Intervention group		Control group	
		n	Median (25th–75th) or %	n	Median (25th–75th) or %
Fruit and vegetables (g/d)	Baseline	691	204.6 (119.1–337.0)	689	191.5 (104.1–304.2)
	First follow-up	618	176.7 (111.0–274.3)	612	164.4 (99.0–247.8)
	Second follow-up	546	150.6 (101.2–248.1)	532	153.1 (48.6–181.9)
Added sugar (g/d)	Baseline	691	68.2 (48.1–92.7)	689	68.9 (84.2–240.4)
	First follow-up	618	58.1 (42.7–77.5)	612	60.3 (44.6–82.9)
	Second follow-up	546	57.1 (41.7–75.2)	532	62.8 (42.9–85.0)
Total fat (E %/d)	Baseline	691	25.5 (22.0–29.0)	689	25.3 (21.6–29.2)
	First follow-up	618	25.5 (21.7–29.3)	612	26.3 (22.4–30.2)
	Second follow-up	546	25.9 (22.1–30.5)	532	26.2 (22.5–30.5)
Unhealthy snacking^b^(g/d)	Baseline	691	94.5 (27.5–220.0)	689	97.5 (40.0–258.0)
	First follow-up	618	63.8 (0.00–184.6)	612	81.5 (12.6–204.6)
	Second follow-up	546	60.0 (0.00–180.0)	532	98.1 (16.3–233.8)
Unhealthy snacking at school (% of consumers)	Baseline	691	18.4	689	20.6
	First follow-up	618	18.1	612	31.4
	Second follow-up	546	26.5	532	32.3
Breakfast intake (% of consumers)	Baseline	691	78.4	689	75.6
	First follow-up	618	79.3	612	77.3
	Second follow-up	546	74.0	532	80.8
Waist circumference (cm)	Baseline	691	68.9 (9.0)	692	68.3 (8.1)
	First follow-up	615	68.3 (8.0)	607	69.4 (7.6)
	Second follow-up	543	71.1 (8.4)	530	71.2 (7.9)

^a^Summary statistics using all the available individual data at baseline and follow-up points adjusted for the cluster design

^b^Consumption of unhealthy foods eaten during snack time; i.e. table sugar, sweets, salty snacks, fast food, soft drinks and packaged food

The sample size retained for analysis of all the dietary intake outcomes included 1046 adolescents in 20 schools; 538 (78% of the sample at baseline) in the intervention and 508 (74% of the sample at baseline) in the control group. Sample sizes for the other outcomes differed slightly (Fig. 1) but the attrition rate was similar for both groups (22 and 20% for the intervention and control group respectively, *P* = 0.55). No significant differences in primary and secondary outcomes at baseline were found between participants who dropped out and those retained for analysis.

### Overall effect

The effect of the program after 28 months is presented in Table 4. Participants from the intervention group consumed lower quantities of unhealthy snacks (−23.32 g; 95% CI: −45.25,1.37) and less added sugar (−5.66 g; 95% CI: −9.63, 1.65) at the end of the trial. Daily fruit and vegetable intake decreased in both the intervention and control groups compared to baseline (Table 3), but the decrease was 23.88 g (95% CI: 7.36, 40.40) lower among the intervention group. Waist circumference (−0.84 cm; 95% CI: −1.68, 0.28) was lower in the intervention group at the end of the program (Table 4).

**Table 4:** Intervention effect at the end of the intervention and by intervention stage

	n	Effect at the end of the intervention (28 months)^a^			Effect after stage 1 (17 months)^b^		Effect after stage 2 (11 months)^b^		
		Diff^c^ (95% CI)	*P* ^d^	ICC^e^	Diff^c^(95% CI)	*P* ^d^	Diff (95% CI)	*P* ^d^	ICC^f^
Added sugar (g/d)	1046	−5.66 (−9.63;-1.65)	0.006	0.36	−2.72 (−6.97; 1.36)	0.20	−4.07 (−8.47; 0.44)	0.07	0.35
Fruit and vegetables (g/d)	1046	23.88 (7.36; 40.40)	0.005	0.26	29.2 (10.4; 47.6)	0.002	−13.4 (−37.4; 10.2)	0.27	0.26
Unhealthy snacking (g/d)^g^	1046	−23.32 (−45.25;-1.37)	0.04	0.16	−9.35 (−32.1; 13.4)	0.42	−18.5 (−44.8; 7.81)	0.17	0.17
Unhealthy snacking at school (PD^h^)	1046	−0.03 (−0.08; 0.06)	0.49	0.10	−0.15 (−0.20;-0.08)	<0.001	0.11 (0.08; 0.50)	0.005	0.11
Breakfast intake (PD^h^)	1046	−0.03 (−0.06; 0.03)	0.39	0.42	0.03 (−0.005; 0.085)	0.11	−0.07 (−0.13;-0.02)	0.011	0.44
Fat (E%/d)	1046	−0.45 (−1.20; 0.31)	0.25	0.22	−0.51 (−1.36; 0.24)	0.17	0.11 (−0.77; 1.10)	0.74	0.21
Waist circumference (cm)	1079	−0.84 (−1.68;-0.28)	0.005	0.77	−1.68 (−2.38;-1.02)	<0.001	0.66 (−0.07; 1.21)	0.08	0.78

^a^Results obtained from mixed effects models accounting for the effects of clustering by individual and school. These models were adjusted for sex, UBN and differences at baseline (excluding the treatment group main effect) [35]

^b^Results obtained from regressions spline mixed effects models. One knot was defined at the mean time of the first follow up data collection (17 months)

^c^Mean differences

^d^ *P* value

^e^Intraclass correlation coefficient of mixed effects models

^f^Intraclass correlation coefficient of regression spline mixed effects models

^g^Consumption of unhealthy foods eaten during snack time; i.e. table sugar, sweets, salty snacks, fast food, soft drinks and packaged food

^h^Proportion difference

### Effect according to intervention stage

The effect was higher during the first stage (Table 4). The effect on fruit and vegetables intake (29.2 g; 95% CI: 10.4, 47.6) and on waist circumference (−1.68 cm; 95% CI: −2.38, 1.02) was in favor of the intervention group and reached statistical significance only after stage one (Table 4). The proportion of consumers of unhealthy snacks prepared at school decreased by 15% (95% CI: −20.0,-8.0) after stage one, but increased by 11% (95% CI: 8.0, 50.0) after stage two in the intervention schools when compared with the control schools. During stage two, the percentage of breakfast consumers also decreased in the intervention schools (−7%; 95% CI: −13.0, −2.0) compared with the control group. On the other hand, the intake of unhealthy snacks decreased largely at stage two but did not reach statistically significance at any stage (−9.35 g; 95% CI: −32.1, 13.4 at stage one and −18.5 g; 95% CI: −44.8, 7.81 at stage two).

### Dose and reach

Dose and reach are reported in Table 5. While 99% of the classes were delivered in stage one, this percentage decreased to 91% in stage two (Table 5). This decrease is explained by the fact that half of the participants reached high school; in the Ecuadorian school system, at high school, adolescents are distributed into different specializations according to their preferences, the last implies that more classrooms had to be included to involve all the sampled adolescents. A higher proportion of schoolteachers implemented the classes themselves in stage one (70%) compared to stage two (31%).

**Table 5:** Dose and Reach of ACTIVITAL by intervention stage

Intervention component	Process evaluation element	Output	Overall (%)	Stage 1 (%)	Stage 2 (%)
The curriculum-based component	Dose delivered	% of classes delivered	94	99	91
	Reach	% of classes delivered by school teachers	46	70	31
The environment-based component/Food kiosks staff workshops	Dose delivered	% of workshops delivered	100	100	100
	Reach	% of schools that attended to at least 80% of the workshops	65	70	60
The environment-based component/Parental workshops	Dose delivered	% of workshops delivered	100	100	100
	Reach	% of parents reached	15	20	11

All the workshops with the food kiosks staff and with parents were performed in both intervention stages. In total, 70% of the schools attended at least 80% of the food kiosks staff workshops at stage one and 60% at stage two. One private school was absent in all the workshops as the school owner operated the food kiosk and considered that the nutritional quality was adequate. Similarly, more parents attended the workshops in stage one vs. stage two (20% vs. 11%).

### Sensitivity analysis

The overall intervention effect did not change considerably after imputing missing values, the effect on unhealthy snacks was no longer significant (*P* = 0.092) and the coefficient decreased from −23.3 to −18.3 g/day (95% CI: 39.5, 2.54). The effect on fruit and vegetables (20 g; 95% CI: 2.24, 37.2), added sugar (−6.7 g; 95% CI: −0.97,-12.5) and waist circumference (−1 cm; 95% CI: −1.64,-0.39) remained significant and with similar coefficients.

## Discussion

The ACTIVITAL trial positively influenced unhealthy snacking, added sugar intake and waist circumference while attenuating the decrease in fruit and vegetables intake in the intervention group. This study adds value to the current literature as it provides a comprehensive school-based intervention designed by means of a theoretical and participative approach able to target multiple populations and risk factors among adolescents living in an Andean upper middle-income country.

At the end of the intervention, fruit and vegetable intake decreased in both the intervention and control groups, but this decrease was lower in the intervention group. Although the intervention was unable to increase the fruit and vegetable intake, it effectively attenuated the decrease of fruit and vegetable intake during adolescence. Still, the intake in the intervention group in the present study remained below the recommended intake of 400 g of fruits and vegetables per day [37] and the effect is lower compared to other school-based health promotion programs in this age group [38, 39]. However, the latter programs, included fruit and vegetables as the only outcome.

We report a decrease of 23 g/d in unhealthy snack intake and a decrease of 15% in the proportion of consumers of unhealthy snacks prepared at school in the intervention group after the first stage. Previous research has demonstrated that multicomponent programs involving parents, aiming at the food sources both in and outside of school and focusing on a variety of unhealthy food items seem to be less likely to fail [40]. The decrease in the fraction of consumers of unhealthy food at school during stage one suggests improvements in the food prepared at school as it concurs with a higher staff response.

Our reductions of 1 cm of waist circumference is comparable to previous studies [41]. Diets high in fruit and vegetables, low in fast food and soda, and improvements in physical fitness were previously found inversely correlated with waist circumference. The ACTIVITAL trial also had important effects on both physical activity and physical fitness [21]. The proportion of adolescents reaching the recommended moderate to vigorous physical activity decreased less in the intervention group, the proportion of adolescents spending more than 3 h in front of a screen decreased in the intervention group during weekdays, performance on the vertical jump test was better among the intervention participants, and finally, participants in the control group performed worse on the speed shuttle run test [21].

The effect on fruit and vegetable intake, unhealthy snacking and waist circumference was larger during stage one of the intervention. Differences in program implementation between stage one and two can explain this. In contrast to stage two, (i) participatory workshops were performed, (ii) dose and reach were higher (iii) and more workshops with the food kiosks staff were implemented during stage one. Previous studies have shown how participatory approaches [42] and parental support are important factors for school-based health promotion [43]. The low parental response could be the consequence of the lack of parents’ recognition of their responsibility in their children’s eating behavior according with qualitative data obtained in the same population [24]. Future studies should identify the most effective strategies to involve parents in diet-oriented health promotion programs implemented in LMICs. Previous reports from high-income countries have documented the difficulty of reaching parents even after adapting interventions to their requirements [44]. The latest research suggests that there is still insufficient evidence on how to engage and reach parents in these kinds of programs.

Cluster randomized controlled trials should have both internal and external validity to enable generalizability [45]. Although our sample size retained for analysis was smaller than expected, there are several reasons to conclude that ACTIVITALs’ internal validity was fairly acceptable: (i) analysis of missing values did not provide different results, (ii) outcome differences between the dropouts and the sample retained for analysis were small, (iii) the cluster nature of the study was considered in both the sample size calculations and data analyses, and, (v) the participants in the intervention and the control group were selected at random.

External validity could be evaluated by the adoption, i.e. the extent to which the setting is representative to the wider population, and the evaluation of the implementation of the program [45]. Regarding adoption, the findings of ACTIVITAL are mainly applicable to urban areas. The program may not be effective among rural adolescents as the needs assessment showed that determinants of healthy eating and physical activity were substantially different between both areas [24, 46]. Even though some urban schools were not considered for randomization in the pair-matching process because they either had very few students or an unavailable matching pair, this selection does not influence the adoption of the program in the urban area. In recent years, the Ecuadorian school system was reformed. Currently, all the schools have become co-ed., and an important proportion of the small schools have closed [47].

We acknowledge a number of limitations. The 24-h recalls were applied only during weekdays. Although the average of two recalls is appropriate to estimate mean group intake [29], it does not allow estimating usual individual intake and the proportion of individuals at risk of inadequate intake [29]. The main purpose of the study however, was to assess differences between the intervention vs. control group. Data on differences between the adolescents who agreed to participate and those who did not was not collected. The causes of the differential effect between stage one and two remain to be confirmed using an effectiveness study comparing a highly controlled intervention with an intervention implemented in usual school conditions [48]. This research would clarify if the attenuated effect is the consequence of a relapse of the program or the consequence of the lack of control in habitual conditions.

## Conclusions

ACTIVITAL had positive effects on dietary risk factors for NCDs, i.e. fruit and vegetable intake and the consumption of unhealthy food items during snacking. Although still below the nutritional recommendations, the combined effect of the intervention is encouraging and promising [4, 38]. It suggests that school-based interventions can address various risk factors simultaneously in adolescents from LMICs. The program strategies must be implemented at the national level by collaboration between the academia and policy makers to assure impact at larger scale.

## Abbreviations

AIC: Akaike Information Criteria
BIC: Schwartz’s Bayesian Information Criteria
CPPE: Comprehensive and Participatory Planning and Evaluation
IM: Intervention mapping
NCDs: Non-communicable diseases
UBN: Unsatisfied basic needs
